# Coordinated Concentration Changes of Transcripts and Metabolites in *Saccharomyces cerevisiae*

**DOI:** 10.1371/journal.pcbi.1000270

**Published:** 2009-01-30

**Authors:** Patrick H. Bradley, Matthew J. Brauer, Joshua D. Rabinowitz, Olga G. Troyanskaya

**Affiliations:** 1 Lewis-Sigler Institute for Integrative Genomics, Princeton University, Princeton, New Jersey, United States of America; 2 Department of Molecular Biology, Princeton University, Princeton, New Jersey, United States of America; 3 Department of Chemistry, Princeton University, Princeton, New Jersey, United States of America; 4 Department of Computer Science, Princeton University, Princeton, New Jersey, United States of America; University of Tokyo, Japan

## Abstract

Metabolite concentrations can regulate gene expression, which can in turn regulate metabolic activity. The extent to which functionally related transcripts and metabolites show similar patterns of concentration changes, however, remains unestablished. We measure and analyze the metabolomic and transcriptional responses of *Saccharomyces cerevisiae* to carbon and nitrogen starvation. Our analysis demonstrates that transcripts and metabolites show coordinated response dynamics. Furthermore, metabolites and gene products whose concentration profiles are alike tend to participate in related biological processes. To identify specific, functionally related genes and metabolites, we develop an approach based on Bayesian integration of the joint metabolomic and transcriptomic data. This algorithm finds interactions by evaluating transcript–metabolite correlations in light of the experimental context in which they occur and the class of metabolite involved. It effectively predicts known enzymatic and regulatory relationships, including a gene–metabolite interaction central to the glycolytic–gluconeogenetic switch. This work provides quantitative evidence that functionally related metabolites and transcripts show coherent patterns of behavior on the genome scale and lays the groundwork for building gene–metabolite interaction networks directly from systems-level data.

## Author Summary

Metabolism is the process of converting nutrients into usable energy and the building blocks of cellular structures. Although the biochemical reactions of metabolism are well characterized, the ways in which metabolism is regulated and regulates other biological processes remain incompletely understood. In particular, the extent to which metabolite concentrations are related to the production of gene products is an open question. To address this question, we have measured the dynamics of both metabolites and gene products in yeast in response to two different environmental stresses. We find a strong coordination of the responses of metabolites and functionally related gene products. The nature of this correlation (e.g., whether it is direct or inverse) depends on the type of metabolite (e.g., amino acid versus glycolytic compound) and the kind of stress to which the cells were subjected. We have used our observations of these dependencies to design a Bayesian algorithm that predicts functional relationships between metabolites and genes directly from experimental data. This approach lays the groundwork for a systems-level understanding of metabolism and its regulation by (and of) gene product levels. Such an understanding would be valuable for metabolic engineering and for understanding and treating metabolic diseases.

## Introduction

Cellular metabolism—the process by which nutrients are converted into energy, macromolecular building blocks, and other small organic compounds—depends upon the expression of genes encoding enzymes and their regulators. Well-characterized transcriptional regulatory circuits such as the *lac* and *trp* operons in *E. coli* and the galactose utilization system in *S. cerevisiae* illustrate how the concentration of metabolites such as tryptophan or galactose can modulate gene expression. In addition, changes in gene expression can lead to increases or decreases in the concentrations of enzymes and regulatory proteins, thereby affecting concentrations of intracellular metabolites. While individual cases of mutual regulation by metabolites and gene products have been and continue to be described, identifying the full scope of these interactions is important for improving rational control of metabolism to meet therapeutic and bioengineering objectives. Clinical scientists, for instance, may be interested in developing novel treatments that control blood glucose levels in diabetic patients, or that fight cancer by disrupting metabolism in tumor cells. This line of inquiry is also relevant to bioengineers seeking to increase the production of small molecules (such as biofuels or flavor molecules) by knocking out or overexpressing individual genes.

The simultaneous measurement of metabolite and transcript concentrations is one method that has begun to show promise for identifying gene products and small molecules involved in the same biological processes [1]. A number of studies [2]–[6] have followed the behavior of specific secondary metabolites of interest such as volatile signaling molecules [4] or compounds with pharmaceutical properties [3], as well as transcripts, in response to genetic or biochemical perturbations. The further refinement of high-throughput experimental technologies such as mass spectrometry has enabled recent studies to measure many functional classes of metabolites together with a large proportion of the transcriptome [7]–[14]. For example, one recent ground-breaking study collected extensive data on metabolite, protein, and transcript levels in *E. coli* following the disruption of genes in primary carbon metabolism or changes in growth rate, and concluded that metabolite concentrations tended to be stable with respect to these perturbations [15]. Another study [12] compared transcript and metabolite concentrations in *S. cerevisiae* under two different growth conditions, and using a novel computational method in which known metabolic pathways were divided into smaller pathways termed “reporter reactions,” the authors observed that when two different growth conditions were compared, the majority of the reporter reactions showed changes in transcript concentrations, with fewer revealing significant alterations in metabolite levels. Such methods, which make inferences based on comprehensive reconstructions of biochemical pathways in an organism, represent valuable tools for analyzing metabolomic and transcriptional data together. However, there is still a need for approaches that are designed to answer the problem of identifying novel interactions between specific gene products and metabolites that include both enzymatic and regulatory relationships.

Of prime importance to the problem of finding gene–metabolite relationships from data is the question of whether functionally-related metabolites and transcripts do indeed show coherent patterns of concentration changes that can be used to make valid predictions. Studies aimed at addressing this question have relied on computing correlation coefficients between profiles of transcript and metabolite concentrations, which can then be ranked [7] or used to co-cluster the metabolomic and transcriptomic data [8]. However, it is possible that other types of regulation, such as post-translational protein modifications and feedback inhibition, could be more predominant in the aggregate than transcriptional regulation [11]. Accordingly, a major limitation with these computational techniques is that the extent to which transcripts and metabolites are co-regulated is not known. The proportion of strong gene–metabolite correlations that are due to chance or indirect effects, as opposed to enzymatic or regulatory relationships, has also not been determined by previous investigations.

In part due to these concerns, previous work has come to contradictory conclusions about the extent of coordination between metabolite and transcript concentrations. Some qualitative evidence has been provided for the claim that transcripts and metabolites are substantially co-regulated [8],[9],[16], including the comparison of clustering patterns in each data set [8], and examples of coherent correlations between biosynthetic enzymes and their products [9]. In contrast, other studies contend that transcript and metabolite profiles tend to behave differently [10], and some have argued that correlative approaches are not specific enough to draw conclusions about which genes and metabolites are functionally related (such that the expression of a gene product controls the concentration of a metabolite, or vice versa) [11],[17].

Indeed, observed correlations within metabolic networks often confound straightforward interpretations. Metabolic networks, unlike transcriptional or protein-interaction networks, consist of molecular species which chemically interconvert. As a result, metabolites that are only distantly related in terms of the underlying pathways can show high levels of correlation [18]. This is especially true in the case of global perturbations (e.g., nutrient starvation, diurnal cycles) which affect many different branches of metabolism at once [19]. It is therefore likely that the interpretation of correlations between transcript and metabolite concentrations will depend on contextual factors, such as the branch of metabolism being studied or the experimental perturbation under which the correlations were observed.

In order to examine these questions further, we conducted a systems-level investigation of the metabolome and transcriptome of *S. cerevisiae*, in which we measure the dynamic responses of metabolites and transcripts to two nutrient deprivations. We examine whether transcripts and metabolites are co-regulated in general, and demonstrate the existence of a strong trend for correlated genes and metabolites to participate in related biological processes. We also demonstrate that the correlations observed for related gene–metabolite pairs are dramatically different depending on the type of metabolite and the perturbation to which the cells are subjected, and we develop a Bayesian algorithm capable of accounting for these dependencies. When applied to our experimental data, this algorithm makes gene–metabolite interaction predictions that are significantly more precise and complete than those made by correlation alone.

## Results

Transcript levels (Dataset S1, GEO accession number GSE11754) were measured via microarray following the induction of carbon starvation (glucose removal) or nitrogen starvation (ammonium removal) at 0, 10, 30, 60, 120, 240, and 480 minutes post-induction. These data complement a previously-published study that measured metabolites in *Saccharomyces cerevisiae* using liquid chromatography–tandem mass-spectrometry (LC-MS/MS), under the same experimental conditions [20]. Both metabolite and transcript samples were collected utilizing a filter-culture approach, which allows the rapid modification of the extracellular environment and fast quenching of intracellular metabolism and transcription 20,21.

### Singular Value Decomposition and Enrichment Analysis Reveal Substantial Coregulation between Transcription and Metabolism

The extent to which transcripts and metabolites show coordinated behavior in response to environmental perturbations remains an open question. It has been observed that metabolite data and transcript data cluster in similar ways [8], yet other studies have noted marked differences in the temporal dynamics of the metabolic and transcriptional responses [10]. Previous systems-level analyses have not presented quantitative evidence either for or against the similarity of the transcriptional and metabolic responses as a whole. To investigate this question, we used singular value decomposition to mathematically extract the signals in the transcriptional and in the metabolic data, and then tested how well these signals were correlated to each other.

**Figure 1:**
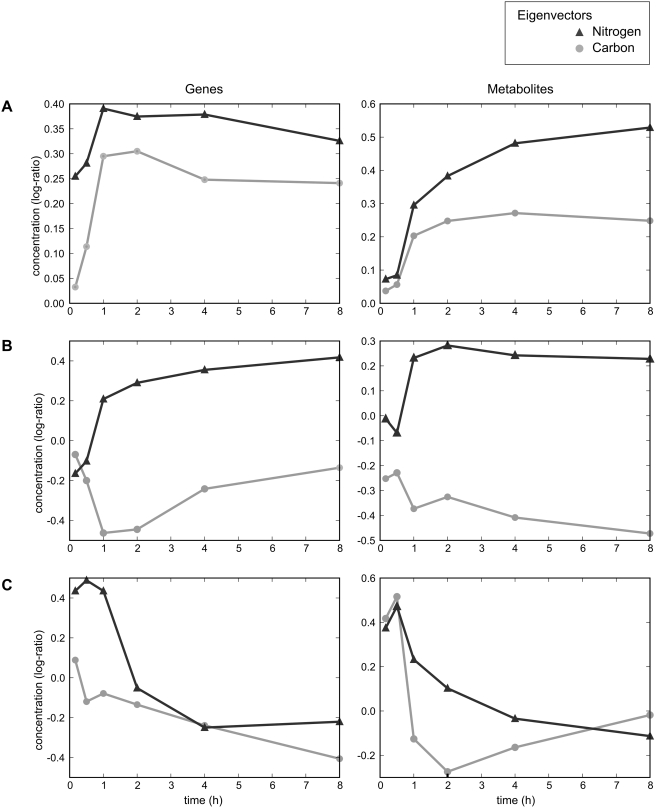
Singular value decomposition reveals coordination between transcriptional and metabolic responses to carbon and nitrogen starvation. Eigenvectors of the transcription (left) and metabolite (right) concentration data sets were calculated. Each eigenvector is composed of a characteristic response under carbon starvation (“carbon”, in light gray circles) and under nitrogen starvation (“nitrogen”, in dark gray triangles). For each gene eigenvector on the left, the corresponding metabolite eigenvector is plotted on the right. The corresponding eigenvectors correlated significantly, with p-values of (A) 6.9×10^−3^, (B) 2.2×10^−4^, and (C) 2.1×10^−2^. For the transcript data, the percent information explained by each eigenvector was (A) 46%, (B) 13%, and (C) 9%. For the metabolite data, the percent information explained was (A) 33%, (B) 20%, and (C) 10%.

Singular value decomposition (SVD) of the gene expression data and of the metabolite data shows that the dominant metabolite abundance patterns are closely aligned with the corresponding transcript abundance patterns (Figure 1). The first eigenvector for both genes and metabolites corresponds to a roughly monotonic starvation response that is similar across carbon and nitrogen deprivation. The second eigenvector is consistent with a nutrient-specific response, and exhibits opposite directionality between carbon and nitrogen deprivation. The third eigenvector represents a difference in dynamics between carbon and nitrogen starvation. Since neither the eigenvectors found by SVD nor the correlation analysis is sensitive to the absolute scale of each pattern in the transcript and metabolite data, the similarities described above are due to the response dynamics, and do not imply similar magnitudes of the responses. However, the magnitudes of the responses that we observed in the transcriptional and the metabolomic data appear to be comparable: the root-mean-squared fold change from the zero timepoint was 3.3-fold for metabolites and 3.1-fold for transcripts. This analysis supports the conclusion that metabolite and transcript concentrations change in quantitatively similar manners following nutrient starvation. Thus, although metabolism and transcription operate on different time scales, in the present study these two processes can be directly compared without explicitly accounting for such a temporal difference.

This result enabled us to ask whether the transcripts and metabolites that show similar dynamics tend to be biologically related: although instances of relationships between the concentrations of metabolites and related biosynthetic enzymes have been described [9],[16], other systems-level studies have noted that the majority of individual gene–metabolite correlations that they observed had no direct interpretation [11]. In order to investigate whether a trend in fact exists for metabolites involved in a certain biological process to show coordinated response patterns with related genes, we conducted a statistical enrichment analysis covering multiple metabolite classes (Materials and Methods). In this analysis, the metabolites that we measured were divided into four broad classes according to their functional role: (a) glycolysis and pentose-phosphate pathway compounds, (b) TCA cycle compounds, (c) amino acids, and (d) biosynthetic intermediates. For each of these classes, a list of associated genes was assembled, such that if a gene was significantly correlated to a metabolite belonging to a particular class, then that gene was considered to be associated with that metabolic class. Significance of correlation was assessed empirically via permutation test and corrected for multiple hypotheses, setting the false discovery rate at 0.01. To find which functions were statistically over-represented in these lists of associated genes, we then performed Gene Ontology term enrichment analysis, using the hypergeometric distribution to obtain p-values which were then Bonferroni-corrected. We selected the Gene Ontology (GO) to perform this enrichment since it has annotations for *S. cerevisiae* that encompass not only enzymes but also regulatory proteins, and since the ontology extends beyond metabolism to cover a wide range of other biological processes such as protein translation and the cell cycle. The full list of enriched biological processes is shown in Table 1.

**Table 1:** Complete results of Gene Ontology (GO) term enrichment for each metabolite class: glycolysis and pentose-phosphate pathway compounds (G/PPP), amino acids (AA), TCA cycle intermediates (TCA), and biosynthetic intermediates (BSI).

Class	GO Term	p-Value	Class	GO Term	p-Value
**G/PPP**	Cell cycle	5.49×10^−5^	**AA**	Protein biosynthesis	<10^−12^
	Mitotic cell cycle	1.39×10^−4^		Nitrogen compound metabolism	<10^−12^
	Protein amino acid N-linked glycosylation	6.17×10^−4^		Biosynthesis	<10^−12^
	Sister chromatid segregation	1.24×10^−3^		Amine metabolism	<10^−12^
	Chromosome segregation	1.30×10^−3^		Cellular biosynthesis	<10^−12^
	Mitotic sister chromatid segregation	4.89×10^−3^		Macromolecule biosynthesis	6.85×10^−12^
	Ergosterol biosynthesis	8.68×10^−3^		Amino acid and derivative metabolism	2.15×10^−7^
	Ergosterol metabolism	8.68×10^−3^		tRNA aminoacylation for protein translation	7.28×10^−7^
	M phase	1.71×10^−2^		Amino acid activation	7.28×10^−7^
	Lipid biosynthesis	2.99×10^−2^		tRNA aminoacylation	7.28×10^−7^
	Mitosis	3.72×10^−2^		Translation	1.71×10^−6^
	Allantoin metabolism	3.94×10^−2^		Amino acid metabolism	2.37×10^−6^
	Allantoin catabolism	3.94×10^−2^		Organic acid metabolism	9.59×10^−6^
	Heterocycle catabolism	3.94×10^−2^		Carboxylic acid metabolism	9.59×10^−6^
	M phase of mitotic cell cycle	4.14×10^−2^		Lipid biosynthesis	2.53×10^−5^
	Protein amino acid glycosylation	4.75×10^−2^		Ergosterol biosynthesis	1.03×10^−4^
	Biopolymer glycosylation	4.75×10^−2^		Ergosterol metabolism	1.03×10^−4^
	Steroid biosynthesis	4.86×10^−2^		Steroid biosynthesis	2.23×10^−4^
	Sterol biosynthesis	4.86×10^−2^		Sterol biosynthesis	2.23×10^−4^
**BSI**	Nitrogen compound metabolism	4.75×10^−5^		Cellular macromolecule metabolism	6.41×10^−4^
	Lipid biosynthesis	1.19×10^−4^		Cellular physiological process	8.65×10^−4^
	Amine metabolism	2.45×10^−4^		Primary metabolism	1.44×10^−3^
	Biosynthesis	2.14×10^−3^		Sterol metabolism	2.10×10^−3^
	Amino acid and derivative metabolism	2.31×10^−3^		Cellular process	2.75×10^−3^
	Amino acid metabolism	6.99×10^−3^		Cellular protein metabolism	2.75×10^−3^
	Ergosterol biosynthesis	9.47×10^−3^		Physiological process	2.76×10^−3^
	Ergosterol metabolism	9.47×10^−3^		Steroid metabolism	2.89×10^−3^
	Organic acid metabolism	1.64×10^−2^		Alcohol metabolism	4.13×10^−3^
	Carboxylic acid metabolism	1.64×10^−2^		Cellular metabolism	4.30×10^−3^
	tRNA aminoacylation for protein translation	4.28×10^−2^		Metabolism	9.60×10^−3^
	Amino acid activation	4.28×10^−2^		Regulation of translation	1.57×10^−2^
	tRNA aminoacylation	4.28×10^−2^		Amine biosynthesis	1.65×10^−2^
	Cellular lipid metabolism	4.91×10^−2^		Nitrogen compound biosynthesis	1.65×10^−2^
**TCA**	Tricarboxylic acid biosynthesis	4.96×10^−2^		Regulation of protein biosynthesis	2.06×10^−2^
				Lipid metabolism	2.66×10^−2^
				Cellular lipid metabolism	4.03×10^−2^

Despite the complexity of the interplay between metabolism and transcription and complicating factors such as post-translational regulation, we found a strikingly logical and biologically relevant relationship between classes of metabolites and the types of gene products to which they were highly correlated. For example, the single significant enrichment result for TCA cycle compounds is the biological process “tricarboxylic acid cycle intermediate metabolism” (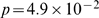). Additionally, the gene products correlated to the amino acid metabolite category are enriched for “amino acid metabolism” (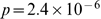) and “tRNA aminoacylation” (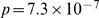). Transcripts correlating with biosynthetic intermediates are enriched for “biosynthesis” (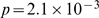), among other processes, and the glycolysis and pentose-phosphate pathway compounds are enriched for “protein amino acid N-linked glycosylation” (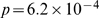). Not all terms show a direct relationship to the metabolite class for which they are enriched: except for the TCA cycle compounds, the profiles of metabolites in every class appear to be correlated to transcripts involved in lipid, ergosterol, and steroid metabolism, a result whose functional relevance has yet to be determined. Additionally, the profiles of the glycolysis and pentose-phosphate pathway compounds also tend to be highly correlated to the expression of genes involved in mitosis and the cell cycle. This enrichment may relate to the fact that, while yeast cells deprived of nitrogen continue to proliferate and divide over the course of an eight-hour experiment, presumably by catabolizing intracellular nitrogen sources, yeast cells starved for glucose arrest and enter stationary phase almost immediately [20].

### Patterns of Correlation between Genes and Metabolites Depend on the Experimental Condition and the Type of Metabolite

While the above approach is adequate to reveal an overall trend for co-regulation of functionally related genes and metabolites, the nature of the co-regulation could vary depending on the experimental condition and the functional role of the metabolite involved. Furthermore, correlations between genes and metabolites can be of varying strengths, ranging from no correlation to a perfectly linear relationship between transcript concentration and metabolite concentration. These different strengths of correlation can be more or less informative about a gene–metabolite relationship, depending on the circumstance under which they are observed. For example, since amino acids and the enzymes involved in their biosynthesis and catabolism are both likely to be strongly affected by a lack of ammonium, it could be the case that instances of co-regulation between genes and amino acids under nitrogen starvation would be more meaningful than correlations of the same strength observed under carbon starvation.

In addition, correlation can be either positive (as the concentration of the gene rises, the concentration of the metabolite also rises) or negative (“inverse”—as the concentration of one rises, the other falls). The levels of related genes and metabolites could exhibit a positive correlation under one condition while having an inverse relationship or no relationship under another, due to condition-specific differences in regulation. For example, 3-phosphoglycerate (3PG) and phosphoenolpyruvate (PEP) are important in both ATP production and biosynthesis (in which they provide carbon skeletons). 3PG and PEP are known to accumulate during carbon starvation via an allosteric regulatory mechanism that prepares the cell for gluconeogenesis and the metabolism of alternate carbon sources; conversely, their abundances fall under nitrogen starvation [20]. However, many of the enzymes that use the metabolites of lower glycolysis as biosynthetic precursors are repressed under both starvation conditions, perhaps to avoid wasting limited resources. These enzymes include *ILV2* (acetolactate synthase, which catalyzes the first step in isoleucine and valine biosynthesis from pyruvate) and *ARO3* (which catalyzes the first step in aromatic amino acid biosynthesis from PEP and erythrose-4-phosphate). Calculating the correlations of 3PG or phosphoenolpyruvate with genes like *ILV2* or *ARO3* over both experimental conditions would, in effect, average two opposite behaviors: anti-correlation in carbon starvation and positive correlation in nitrogen starvation. There would be no overall correlation, although the behavior could well be consistent with a functional gene–metabolite relationship.

**Figure 2:**
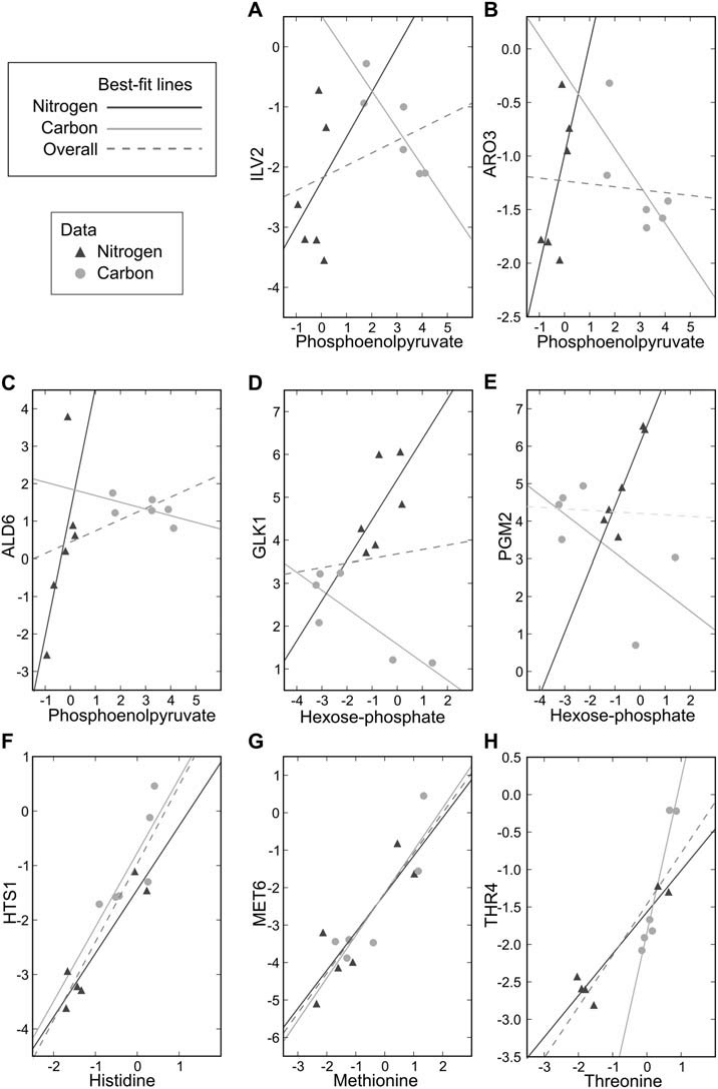
A selection of example scatterplots that demonstrate experimental condition-dependent correlation between metabolites and related genes, motivating the use of a Bayesian algorithm. Metabolite and gene transcript concentration changes are represented as 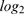 ratios of measurements from starved cells to measurements from unstarved cells. The responses observed under carbon starvation (light gray circles, “Carbon” in legend) are labeled distinctly from the responses under nitrogen starvation (dark gray triangles, “Nitrogen” in legend), but are plotted on the same axes. Solid light gray and dark gray lines are linear best-fits for the responses observed under carbon and nitrogen starvation, respectively; the dashed line is a linear best-fit curve for all data. (A–E) Scatterplots of metabolites from the glycolysis and pentose-phosphate pathway metabolic class versus related genes show an inverse relationship under carbon starvation, but a positive correlation under nitrogen starvation. The dashed line shows that this relationship would be obscured by computing correlation across all data points. *ILV2* catalyzes the first step of isoleucine and valine biosynthesis from pyruvate; *ARO3* catalyzes the first step in aromatic amino acid biosynthesis from PEP and erythrose-4-phosphate; *ALD6*, which also plays a key role in redox metabolism, is involved in the creation of cytosolic acetyl-CoA from pyruvate; *GLK1* phosphorylates glucose to glucose-6-phosphate; and *PGM2* catalyzes the interconversion of glucose-1-phosphate and glucose-6-phosphate. (F–H) Scatterplots of metabolites from the amino acid metabolic class versus related genes, in contrast, show positive correlation in both carbon and nitrogen starvation. Even in this case, however, computing correlation across both conditions can lead to an underestimation of the extent of the relationship (e.g., (H) threonine vs. *THR4*, where although 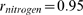 and 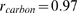, 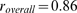). *HTS1* charges (i.e. aminoacylates) the histidinyl-tRNA; *MET6* catalyzes the formation of methionine from homocysteine; and *THR4* converts phosphohomoserine to threonine.

Condition-specific behavior is indeed observed for these gene–metabolite pairs, as well as for the pairs “*ALD6* to phosphoenolpyruvate,” “*GLK1* to hexose phosphate,” and “*PGM2* to hexose phosphate” (Figure 2A–E, in which the concentrations of metabolites belonging to the “glycolysis and pentose-phosphate pathway” class and the concentrations of functionally related gene products are plotted against each other). Ald6p oxidizes acetaldehyde to acetate, and in addition to its key role in redox metabolism [22],[23], is involved in the production of acetyl-CoA from glycolytic end products [24]–[27]. The enzyme Glk1p phosphorylates glucose to glucose-6-phosphate in the first irreversible step of glycolysis [28], and Pgm2p catalyzes the conversion of glucose-1-phosphate to glucose-6-phosphate during the metabolism of alternative carbon sources such as galactose [29]. The metabolite “hexose phosphate” refers to glucose-6-phosphate as well as its isomers (e.g., fructose-6-phosphate, with which glucose-6-phosphate is interconverted), which were not distinguishable in the present LC-MS/MS analysis.

Overall, these glycolytic and pentose-phosphate pathway metabolites show positive correlations (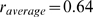) with a number of related genes under nitrogen starvation but negative correlations (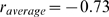) under carbon starvation (Figure 2A–E; representations of the nitrogen starvation data and best-fit lines using expanded x-axes can be found in Figure S2). Computing correlation across both conditions would lead to the erroneous conclusion that no relationship exists between these genes and metabolites (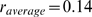). In contrast, for metabolites belonging to the “amino acids” category (Figure 2F–H), related metabolites and genes tend to show strong positive correlations under both conditions: histidine and *HTS1* (the histidine tRNA synthetase), methionine and *MET6* (methionine synthase), and threonine and *THR4* (threonine synthase) exemplify this behavior (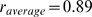). We have therefore developed a Bayesian algorithm capable of automatically learning and exploiting the way in which different signs and strengths of correlation can be suggestive of a functional relationship, depending on the experimental condition and the metabolite class.

### Bayesian Analysis Captures Context-Dependent Patterns of Correlation between Genes and Metabolites

**Figure 3:**
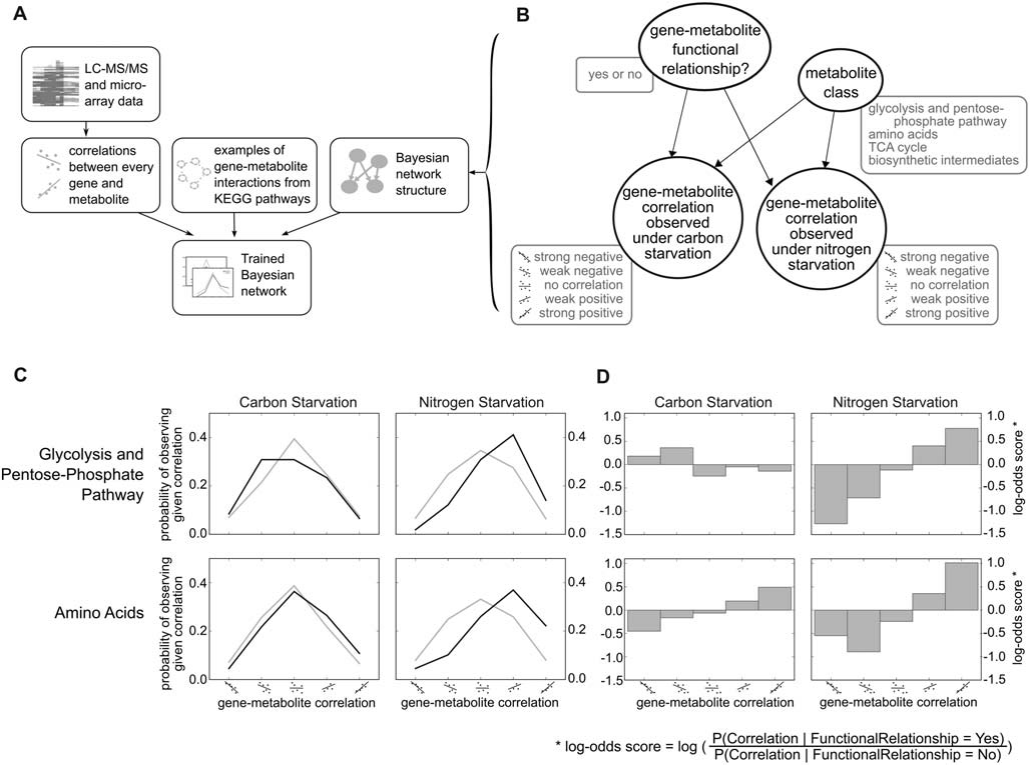
Bayesian network relating gene–metabolite interactions to metabolomic and transcriptomic data and to metabolite class. (A) Overview of Bayesian integration procedure. Transcript and metabolite data were used to compute correlations between genes and metabolites over time under different experimental conditions. These correlations, along with a set of positive and negative examples obtained from KEGG, were used to train a Bayesian network. (B) Structure of the Bayesian network. This four-node network states that the variables corresponding to gene–metabolite correlations observed under either nitrogen or carbon starvation depend on the class of the metabolite involved, and whether or not a functional relationship between the gene and metabolite exists. The rounded boxes by each node represent the possible values that the nodes can take. (C) Conditional probability distributions learned from the experimental data. The parameters of the Bayesian network were computed from the experimental data and the set of positive and negative examples of gene–metabolite functional interactions. The light gray line gives the probability (y-axis) that, given no functional relationship, one would observe a given correlation (x-axis); the dark gray line gives the corresponding probability if given a true functional relationship instead. (D) Conditional probability distributions represented as log-odds scores. The sign of the bar corresponds to whether observing a certain strength and direction of correlation is more likely for a true functional relationship (positive) or for no functional relationship (negative), while the magnitude of the bar corresponds to how much more likely this is.

Bayesian networks [30]–[32] are a general class of graphical probabilistic models. Because they allow the specification of dependencies between quantities of interest, such as relationships observed between genes and metabolites under different conditions, Bayesian networks are well-suited for leveraging such dependencies in order to make specific predictions. In these networks, variables, or “nodes,” are connected by arrows, or “edges,” indicating which variables depend on which others. Each node is parametrized by a conditional probability distribution (CPD), which describes the probability of observing the variable in a certain state, given the states of the variables on which it is dependent (for example, in Figure 3B, “gene–metabolite correlation observed under carbon starvation” is dependent on both “metabolite class” and whether a “gene–metabolite functional relationship” exists).

Our objective in constructing this Bayesian network was to formalize the concept that the strength and direction of correlation observed between a certain gene and metabolite is particular to the experimental perturbation, and depends on the functional class to which the metabolite in question belongs. We also expect to observe different correlations for metabolites and genes that are truly related than we would observe for random, unrelated gene–metabolite pairs. The Bayesian network that we constructed (Figure 3B) therefore consists of four nodes. Two of these nodes correspond to observed correlations calculated from LC-MS/MS and microarray data (“gene–metabolite correlation observed under nitrogen starvation” and “gene–metabolite correlation observed under carbon starvation”); each of these nodes can take one of five different values, depending on the strength and sign of correlation. The other two nodes (“gene–metabolite functional relationship,” which can be yes or no, and “metabolite class,” which can be any of the four metabolite classes enumerated above) correspond to intrinsic attributes of the gene–metabolite pair. To represent the dependencies described above, edges have been drawn from the node representing “functional relationship” and from the node representing “metabolite class” to both of the nodes representing gene–metabolite correlations observed under a specific experimental condition.

Given a set of positive and negative examples, the conditional probability distributions that constitute the parameters of our model can be automatically learned. These distributions are given by 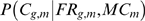 and 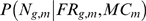, where 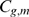 refers to the correlation of gene 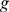 and metabolite 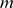 under carbon starvation, 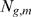 to correlation under nitrogen starvation, 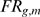 to whether or not a functional relationship exists between gene 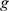 and metabolite 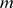, and 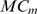 to the class of metabolite 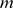. By Bayes' theorem, these class-specific conditional probability distributions (CPDs) are equivalent to the probability that a pair is functionally related given a certain observed level of correlation, normalized by 1) whether that level of correlation is rare or common overall and by 2) whether functional relationships are rare or common overall (i.e., 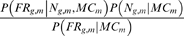 and 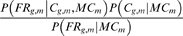). To learn these parameters, we calculated how often different correlations were observed for a set of gene–metabolite pairs known to be either functionally related or unrelated. Positive examples were drawn from genes and metabolites belonging to the same pathway in the Kyoto Encyclopedia of Genes and Genomes (KEGG [33]); negative examples were random gene–metabolite pairs that were not in the positive example set (see Materials and Methods for details).

A key advantage of Bayesian networks, compared to other machine-learning techniques, is that since the parameters are probability distributions, they have a direct meaning which can be informative about the system being modeled. With this in mind, the parameters 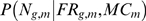 and 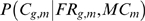 are shown in Figure 3C, for two of the metabolite classes (“glycolysis and pentose-phosphate pathway” and “amino acids”) and all possible values of 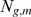, 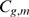, and 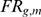. Intuitively, these probabilities capture how likely an observed gene–metabolite correlation would be if the gene–metabolite pair were either related (dark grey) or unrelated (light grey). For example, in the plots on the right-hand side of Figure 3C (nitrogen deprivation data), the distribution for functionally-related pairs is shifted substantially to the right: this indicates that functionally related gene–metabolite pairs tend to be positively correlated under nitrogen starvation.

Another visualization of these conditional probability distributions is shown in Figure 3D. Here, the CPDs are collapsed into a single bar chart for each metabolite class and environmental condition by taking the log-ratio of the CPDs represented by the light and dark lines in Figure 3C. These log-odds scores are given mathematically by 
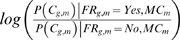
 and 
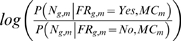
. This visualization is particularly useful because it clarifies whether a particular level of correlation is more likely to be observed for a related gene–metabolite pair (above zero) or for an unrelated pair (below zero). For instance, this figure shows that for amino acids (second row), negative correlations under either condition are more likely to be observed for unrelated gene–metabolite pairs than for pairs where a functional relationship exists. The magnitude of each bar corresponds to how much more probable a particular correlation is for either related or unrelated pairs. For example, in the case of the amino acids, while a positive correlation under either experimental condition suggests a functional gene–metabolite relationship, positive correlation is more informative under nitrogen starvation than it is under carbon starvation.

The values that the network learned for these parameters indicate that the magnitude and direction of correlation between a given gene and metabolite do in fact depend strongly on that metabolite's class, as suggested by Figure 2. For instance, the amino acid methionine and the biosynthetic gene *MET6*, which converts homocysteine to methionine, have a clear functional relationship. Consistent with the parameters learned, methionine and *MET6* exhibit a strong positive correlation under both conditions, especially nitrogen starvation (Figure 2G). In contrast, for glycolysis and pentose-phosphate pathway compounds, while related gene–metabolite pairs do exhibit positive correlations under nitrogen starvation, interacting pairs actually tend to be inversely correlated under carbon starvation. This relationship is typified by *GLK1* and hexose-phosphate (Figure 2D). Additionally, when hexose-phosphate concentrations are plotted against *GLK1* transcript concentrations, it is readily apparent that because hexose-phosphate and *GLK1* are positively correlated under nitrogen starvation but inversely correlated under carbon starvation, they exhibit a very weak relationship when Pearson correlation is computed across both conditions (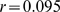).

This pattern of positive correlation under nitrogen starvation and inverse correlation under carbon starvation is also observed for a number of other gene–metabolite pairs in our standard of examples (Figure 2A–E), including phosphoenolpyruvate (PEP) and *ALD6* (Figure 2C). In terms of chemical steps, PEP is linked to *ALD6* indirectly (being first converted to pyruvate by *CDC19* and then to acetaldehyde via pyruvate decarboxylase, the major isozyme of which is *PDC1*). However, PEP, like *ALD6*, is predominantly cytoplasmic, whereas the intermediate species pyruvate and acetaldehyde exist in both cytoplasmic and mitochondrial pools, which could be regulated differently. This suggests that the total cellular concentrations of PEP might be more strongly related to *ALD6* concentrations than would those of the other intermediate species, and furthermore that gene–metabolite pairs that are not directly linked by a single biochemical reaction may still have important functional relationships.

This type of Bayesian integration does not attempt to infer causality between changes in gene and metabolite levels. In certain cases, however, we do have a prior expectation that can explain some of the learned parameters. For example, lack of ammonium under nitrogen starvation likely leads directly to falling amino acid concentrations. Nitrogen starvation also leads to decreased activity of the transcription factor *GCN4* and thus reduced expression of amino acid biosynthetic genes. Although the mechanism is not fully understood, there is evidence that the TOR pathway, which is believed to sense intracellular concentrations of glutamine [34], is responsible for causing reduced translation of *GCN4* via the protein Eap1p [35]. Under carbon starvation, many transcripts may be induced or repressed by a combination of extracellular pathways for the sensing of glucose (via Ras/PKA and Snf3p) and intracellular sensing of hexose-phosphate (potentially mediated by *HXK2*) [36]. While these pathways are elaborate and involve many layers of regulation, it has been observed that during growth without glucose, repression involving *HXK2* and *MIG1* is relieved [37]. In the absence of glucose, we would expect glucose-6-phosphate, fructose-6-phosphate, and FBP levels to drop: since *HXK1* and *GLK1* have been shown to be under the control of *HXK2*-dependent glucose repression [38], this would explain the inverse correlation observed between, for example, *GLK1* and hexose-phosphate.

### Bayesian Integration Finds Specific Gene–Metabolite Interactions outside Our Standard of Examples

**Figure 4:**
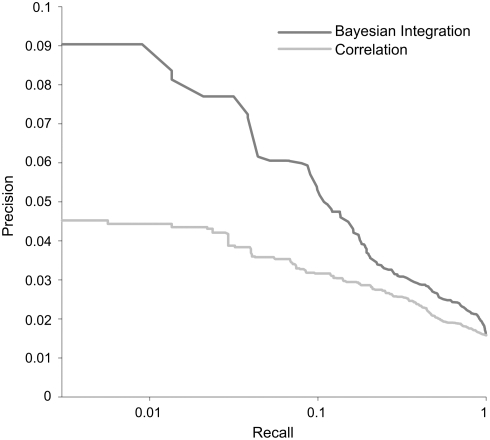
Precision-recall curve (PRC) showing the superior performance of context-sensitive Bayesian integration (dark gray line), as compared to overall strength of gene–metabolite concentration correlation (light gray line), for identifying gene–metabolite functional interactions from transcriptomic and metabolomic data. Recall, or fraction of known positives predicted by the system, is plotted on the X-axis (log scale); precision, or fraction of predictions that are in the training set, is plotted on the Y axis. The Bayesian integration PRC shows greater area under the curve than the correlation PRC, especially in the left-most, highest-confidence regime.

Following parameter learning, we performed inference using the Bayesian network, which assigned to each gene–metabolite pair a confidence score. This score is equal to the posterior probability of a functional relationship, given the metabolite class and the correlations observed in the data (i.e., 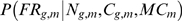). Since this value is continuous between 0 and 1, different cutoffs can be chosen depending on whether a certain application requires more precision (the fraction of pairs above the cutoff that are true positives) or more recall (fraction of total true positives with a score above the cutoff). One way to assess performance that takes this trade-off into account is to plot precision against recall for every possible cutoff, yielding a precision-recall curve (PRC). The same type of PRC can also be generated using the Pearson correlation between metabolite and gene concentrations instead of the gene–metabolite confidence score. We have employed these PRCs to compare the performance of our method relative to simply computing correlation across both experiments (Figure 4).

Given the differences between the parameters learned for distinct perturbations and metabolic classes, we expected that many physiologically relevant, specific gene–metabolite interactions that can be discovered by this Bayesian analysis would be missed by looking only at overall correlation. In agreement with this expectation, when evaluated against our set of known gene–metabolite interactions (using three-fold cross validation to avoid overfitting) and compared to Pearson correlation, Bayesian integration performs significantly better (Figure 4). It is more precise than correlation overall, and reaches twice the precision at the most stringent cut-off (the leftmost end of the curve), which corresponds to the most confidently-predicted gene–metabolite interactions.

To investigate the potential of the Bayesian network to find biologically relevant interactions beyond the set of examples, we searched for support in the scientific literature for the most confident predictions of our network (764 predicted gene–metabolite interactions, excluding those belonging to the example set derived from KEGG), as well as for 250 random gene–metabolite pairs. While many true predictions could be novel and thus unsupported in the literature, we still expect that accurate predictions would be enriched for pairs supported by existing published evidence. Each gene–metabolite pair was scored on four specific criteria (see Materials and Methods). The evaluation was performed blind to whether gene–metabolite pairs were predicted or randomly picked. Of the random pairs, only 1.2% received literature support. In contrast, 9.4% of the highly-predicted pairs were supported by at least one piece of literature evidence, an enrichment of 7.8-fold (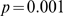 by Fisher's exact test; for contingency table, see Table 2). Whereas no random pair satisfied all four evidence criteria, three predicted pairs did: methionine-*MET3*, methionine-*MET22*, and methionine-*MET10*. These three pairs were not in our gold standard because they participate in the assimilation of sulfur into homocysteine, and although homocysteine is converted in one step to L-methionine, in the KEGG database “sulfur metabolism” does not contain the molecular species “methionine” and is a separate pathway from “methionine metabolism.” Nevertheless, *MET3*, *MET10*, and *MET22* are essential for methionine biosynthesis and the knockouts are methionine auxotrophs. We also found a variety of other genes and metabolites for which there was substantial evidence: e.g., valine-*PDC5* (*PDC5* is involved in the catabolism of valine to isobutyl alcohol [39]), and methionine-*MIS1* (*MIS1* is required for the formylation of the mitochondrial initiator 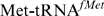 [40]). The full results can be found in Dataset S3. These results suggest that, despite the limited scale of the present work, our approach is capable of generalizing from our training set to find other biologically relevant gene–metabolite interactions.

**Table 2:** Literature support for predicted gene–metabolite interactions.

	Scoring Pairs	Non-scoring Pairs	Total
Random	3	247	250
Predicted	72	692	764

Contingency table representing enrichment of predicted pairs for evidence in literature validation study (by Fisher's exact test, 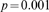). Dataset S3 contains the full results from the literature study.

**Figure 5:**
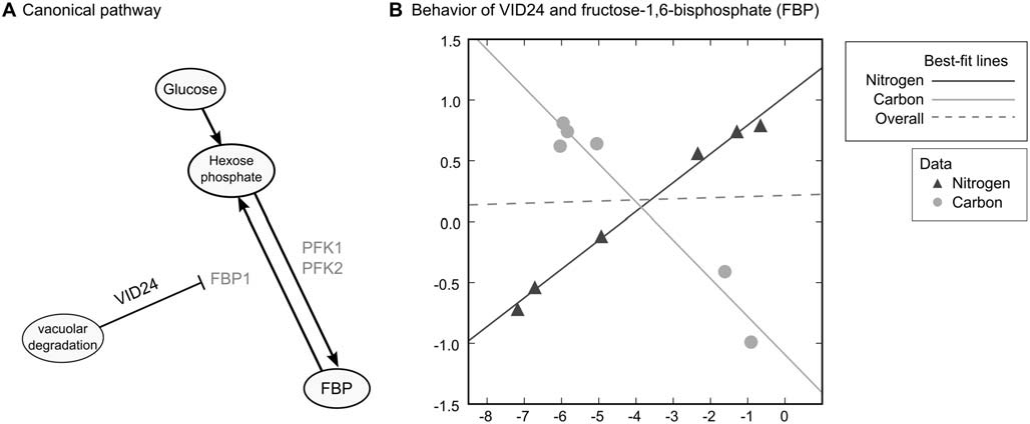
Example of gene regulating the glycolytic–gluconeogenic switch (*VID24*) that we identified as interacting with the key glycolytic metabolite fructose-1,6-bisphosphate (FBP). (A) Schematic of reactions involving FBP and *VID24*. The conversion of FBP to hexose phosphate is catalyzed by fructose-1,6-bisphosphatase (*FBP1*). Vid24p destroys this enzyme by targeting it to the vacuole for destruction. (B) Scatterplot showing the relationship between *VID24* and FBP abundances over carbon starvation (“carbon” in the legend) and nitrogen starvation (“nitrogen” in the legend). As in Figure 2, lines represent linear best-fit curves, calculated separately for each condition (solid lines) or over both conditions (dashed line). *VID24* and FBP are inversely correlated under carbon starvation (light gray), but positively correlated under nitrogen starvation (dark gray), as anticipated for a gene interacting with a glycolytic metabolite. *VID24* was in the top 3% of predictions made for FBP.

A further example of the potential utility of the Bayesian approach is illustrated in Figure 5, in which we describe an interaction identified by Bayesian integration between a metabolite and a protein that regulates enzyme concentrations. This regulatory protein functions as an important part of the system that *S. cerevisiae* has evolved to face a fundamental metabolic challenge: namely, the diauxic shift, during which the cell changes from fermentative to respirative growth. In the first phase of growth on fermentable sugars, *S. cerevisiae* cultures initially grow quickly, metabolizing all the available glucose to ethanol (high ethanol concentrations are toxic to many other microbes, giving *S. cerevisiae* a competitive advantage). This fermentative phase is followed by a second phase of growth in which yeast cells use ethanol as a substrate, and perform oxidative respiration. The switch between these two states involves extensive metabolic and transcriptional remodeling [41]. Chief among the changes induced by the diauxic shift is the shift from using glucose to generate ATP (glycolysis), to using ethanol and ATP to make glucose and the carbon skeletons necessary for biosynthesis (gluconeogenesis). Many of the steps in both glycolysis and gluconeogenesis are readily reversible and are therefore catalyzed by the same enzymes. For this reason, it is imperative that the cell be able to commit to one pathway or the other by controlling the enzymes that are unique to each pathway, as otherwise the cell would waste energy through futile cycles. Accordingly, *S. cerevisiae* has evolved extensive regulatory machinery at the metabolic, transcriptional, and post-transcriptional levels that allows it to successfully negotiate this transition.

One of the key steps of glycolysis is the irreversible conversion of fructose-6-phosphate (F6P) to fructose-1,6-bisphosphate (FBP), catalyzed by phosphofructokinase (the genes *PFK1* and *PFK2*); in gluconeogenesis, the opposite reaction is catalyzed by a separate enzyme, fructose-1,6-bisphosphatase (Fbp1p). A schematic of this pathway is given in Figure 5A. One of the top predictions made by the Bayesian network for the metabolite fructose-1,6-bisphosphate (FBP) was the gene *VID24*, which was not in our gold standard of examples. However, *VID24* is known to play an important regulatory role in governing the gluconeogenetic enzyme Fbp1p: during the switch from gluconeogenesis to glycolysis, Fbp1p is specifically targeted to and degraded in the vacuole in a way that is dependent on *VID24* [42]. This example highlights the promise of Bayesian integration to find relationships that correlation alone would miss. Pearson correlation calculated between *VID24* and FBP across both conditions yields 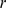 equal to just 0.03. However, as shown in Figure 4B, *VID24* and FBP exhibit an inverse correlation under carbon starvation (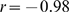) and a strong positive correlation under nitrogen starvation (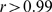). According to the parameters learned by the Bayesian network for the “glycolysis and pentose-phosphate pathway” metabolite class, this behavior is indicative of a gene–metabolite functional relationship with a high likelihood. It is important to note that this interaction was found despite the fact that our study did not explicitly target the diauxic shift, suggesting the capacity of this method to recover diverse functional signals in the data. Moreover, this example shows that interactions can be found not only between genes encoding enzymes and the metabolites they act on, but also between metabolites and proteins that play roles in metabolic regulation.

## Discussion

We have generated paired transcriptional and metabolomic data that capture the dynamic responses to two perturbations over time, and find substantial evidence for the co-regulation of transcripts and metabolites. At a general level, singular value decomposition reveals that the dominant dynamic patterns exhibited by transcript and metabolite concentrations are closely aligned. Functional enrichment demonstrates that metabolites tend to show significant correlations to genes that play roles in related biological processes. Finally, using a Bayesian framework, we are able to find patterns of co-regulation between genes and metabolites that take into account both the experimental context where the correlations are observed, and the functional classification of the metabolite in question.

By analyzing metabolite and transcript data within this framework, we can identify new interactions and detect both direct and indirect regulatory relationships between a broad range of genes and small molecules. For example, we identified a regulatory link between FBP and *VID24*, although they exhibit almost no net correlation across both of the environmental perturbations tested (Figure 5). Additionally, our top predictions also include gene–metabolite relationships that connect metabolism to other key biological processes: for instance, methionine is known to play a unique and important role in the initiation of translation, and indeed two of our top predictions link methionine to *FUN12* and *GCN3*, which are both involved in the formation of the 80S initiation complex that includes 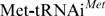 [43]–[45].

This type of Bayesian integration has been shown to outperform conventional correlation-based analyses (Figure 4), and the literature study suggests that we are able to find true gene–metabolite interactions outside of the gold standard. Furthermore, our ability to verify via literature search a substantial fraction of the predicted gene–metabolite pairs (Table 2) implies that Figure 4 markedly underestimates the precision of the Bayesian approach: many of the apparent “false positives” reflect real interactions that were not included in the limited set of positive examples selected from KEGG. These include both real interactions that are already known in the literature, and novel interactions to be verified in follow-up efforts. Identifying such novel gene–metabolite relationships could be used not only to drive further experimentation, but also to contribute to other modeling approaches that rely on extensive knowledge about cellular metabolic networks and their connectivity.

Despite this progress, there is undoubtedly still room for advances to be made in the accuracy of the predicted gene–metabolite interactions. For instance, advances in analytical techniques continue to allow the measurement of larger numbers of known compounds. Although the metabolite classes that we describe in the present work are broadly applicable and cover the majority of primary metabolism, they could also be extended to cover biomolecules that were not measured in the current study, such as lipids or secondary metabolites. Additionally, an increase in measured compounds could allow broader classes such as “biosynthetic intermediates” to be divided into smaller groups like amino acid intermediates or nucleotides, allowing more specific predictions to be made without the risk of overfitting based on a small number of examples. Using a larger number of classes could also help to avoid situations in which a small number of metabolites in a particular class exhibit different behavior from the majority, potentially leading to incorrect predictions for those outlier metabolites. Another area for future development is the gold standard itself, which, although certainly sufficient to make valid predictions, is still incomplete, as shown by the literature study. The gold standard could productively be combined with an extensive curation of the yeast metabolism literature, so that known regulatory as well as enzymatic interactions between genes and metabolites would then be included.

It should also be noted that the current predictions were made on the basis of only two experimental conditions. As interest in the measurement of multiple biomolecule types grows, more paired gene–metabolite data of the type presented here will continue to be published, and we imagine that these data will prove a valuable resource for integration efforts like the present work. Selected data sets that could prove particularly illuminating include metabolome and transcriptome sampling under other elemental starvations, such as phosphate and sulfur. Additionally, since prototrophic yeasts are capable of growth on a variety of carbon and nitrogen sources, monitoring gene and metabolite concentrations under these conditions could be illuminating with respect to both general (e.g., preferred vs. non-preferred nutrient sources, such as ammonium vs. proline) and specific gene–metabolite interactions (e.g., repression or activation of the GAL pathway by galactose).

As compounds from more branches of metabolism can be measured, and as data sets that track multiple biomolecule types in response to perturbations become available for more experimental conditions, analyses that are sensitive to biochemical context are likely to become increasingly critical. This work represents proof-of-concept of the potential of context-sensitive approaches for building networks relating metabolic activity and gene expression directly from experimental data.

## Materials and Methods

### Limitation Experiments on Filters

Cultures of FY4 (a prototrophic, Mat*a* derivative of S288C [46], Princeton strain DBY11069) were grown overnight in liquid minimal media (YNB, see below). After these overnight cultures were set back, 10 mL of early exponential phase culture (Klett 60, 1.5×10^6^ cells/mL) was filtered onto 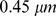 pore-size nitrocellulose filters (82 mm in diameter). The cells (1.5×10^7^ cells with diameter 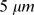) covered 5% of the filter surface. The filters were then placed on minimal media-agarose plates and allowed to grow for 3 h at 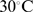, or approximately one doubling on the filter. To initiate the starvation time-course, the filters were transferred from the minimal plates to plates made with media lacking either ammonium (YNB-N, nitrogen deprivation) or D-glucose (YNB-C, carbon deprivation). The filter-culture approach, which allows for both rapid modification of the extracellular environment and rapid quenching of metabolism, is described in detail in previous work [20],[21].

The transcriptome and metabolome were sampled during exponential growth (before switching) and at 10, 30, 60, 120, 240, and 480 minutes following the switch to nitrogen-free or carbon-free media. Measurements of both metabolites and transcripts were collected in parallel. The metabolite measurements and extraction procedures have been previously published [20]. The observed quantitative metabolite concentration changes were verified by an independent experiment that included isotopically-labeled standards of 34 metabolites during the measurement process. This validation demonstrates that the metabolite measurements are robust to potential ion suppression artifacts and experimental noise (see Figure S1 and Brauer et al. [20]).

Experimental controls also demonstrate that the presented metabolomic and transcriptomic data are dominated by biological signal and not by noise. Raw LC-MS/MS data (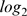 transformed ion counts) for two independent replicates of exponentially growing yeast are plotted in Figure S3. The agreement between the two samples was found to be high (y = 1.03x. 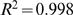). The Lin's concordance coefficient [47], a normalized measure of the distance from the 45° line representing 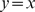, where 0 is non-reproducible and 1 is perfectly reproducible, was 0.98, indicating very high reproducibility.

For the transcriptomic data, two additional negative control replicates were collected by extracting RNA from filter cultures moved to plates containing both a carbon and a nitrogen source (i.e., the same nutrient conditions as before the switch). The median standard deviation in transcript measurements collected from these replicates was found to be 0.099 (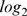 units). In contrast, the median standard deviation for the carbon starvation timecourse was 0.45, and for the nitrogen starvation timecourse 0.46. This demonstrates that the primary source of variability in the presented data is not due to technical or biological noise.

### Transcriptome Sampling

In order to take timepoints of transcription, the filter cultures were submerged in liquid nitrogen and stored at 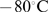. Yeast cells were washed from filters with 10 mL of lysis buffer, and RNA was subsequently extracted using a Qiagen RNEasy kit (QIAGEN, Valencia, CA). Oligo(dT) resin from an Oligotex midi kit (QIAGEN, Valencia, CA) was used to purify the poly(A)+ fraction from the total extracted RNA. cRNA labeled with cyanine (Cy)5- (experimental) or Cy3-dCTP (reference) was then synthesized from 1 to 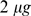 of the poly(A)+ RNA. The transcriptional profiles of yeast cultures at the time of harvest were measured by hybridization of the Cy3- and Cy5-labeled cRNA to an Agilent Yeast Oligo Microarray (V2). The reference sample was the zero-timepoint (taken during exponential growth prior to media switching).

### Normalization of Transcriptome and Metabolome Data

Metabolite levels were normalized by cell dry weight to account for cell growth and division during the time course. As metabolites were roughly evenly-distributed between increasing and decreasing in response to nutrient starvation, no normalization for total metabolome size or total LC-MS/MS signal was required. For transcript levels, cell growth and division was accounted for by loading an approximately equivalent amount of reference and experimental RNA onto each array. This loading also normalized for decreases in total RNA pool size induced by nutrient starvation. Such normalization is useful to enable the identification of specific transcriptional regulatory events, as opposed to changes in the overall level of transcription. To correct for biases in hybridization efficiency between the Cy3 and Cy5-labeled RNA, microarray chip scans were normalized so that the total intensities across all probes in the red and in the green channels were equal.

For the purposes of our analyses, both metabolite and transcript levels were expressed as log base 2 ratios of the zero timepoint. Missing values for the transcriptional data were imputed using KNNimpute [48] with 10 neighbors, discarding any genes having more than 30% missing values; metabolites with missing values were discarded.

### Singular Value Decomposition

Singular value decomposition (SVD) is a process used to elucidate predominant patterns in large data matrices; its applications include image compression and noise reduction. SVD transforms a single data matrix into three matrices: these correspond to (i) the characteristic patterns, or “eigenvectors”; (ii) the amount of information each pattern contributes to the original data set as a whole; and (iii) the weight of each pattern for individual variables. Alter et al. [49] contains a more detailed treatment. Singular value decomposition was performed in MATLAB using the svd command.

To determine the extent of coordination between metabolism and transcription under the conditions tested, we computed the Pearson correlation between the most informative gene patterns (top eigenvectors) and corresponding metabolite patterns. We found that each of the first three gene patterns correlated significantly with the corresponding metabolite patterns, suggesting that similar overall trends were exhibited in both types of data. Significance was established via t-test (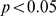).

The root-mean-squared fold change (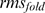) for each of the data types was computed according to the following formula:
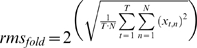
where 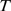 is the number of timepoints (12 for either data type), 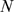 is the number of molecular species measured (5373 for transcripts and 61 for metabolites), and 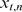 is a particular abundance level observed at timepoint 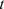 for gene or metabolite 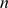 expressed as a log_2_ ratio to time 0. The root-mean-squared fold change was 3.1 for transcripts and 3.3 for metabolites.

### Gene Ontology Enrichment Analysis

For each metabolite 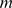 and gene 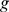 measured, we calculated the Pearson correlation between them:
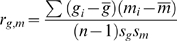
where 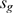 and 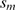 correspond to the sample variance of 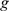 and 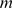, and 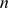 is the sample size (i.e., total number of observations) of 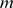 (or 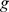).

We then conducted a permutation test, rearranging the columns (i.e., experimental conditions) of the metabolite data matrix 10^4^ times to get bootstrapped p-values for these correlation values, which were then corrected using a false discovery rate of 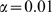 according to the procedure described by Benjamini and Hochberg [50]. The significantly-correlated genes for each metabolite were assembled into lists. We combined all the gene lists for every metabolite in a particular class (TCA cycle, glycolysis and pentose-phosphate pathway, amino acids, or biosynthetic intermediates), yielding four larger gene lists, one for each metabolite class.

The Gene Ontology (GO) [51] is a hierarchical categorization scheme for genes in several organisms, including *S. cerevisiae*. There are three top-level nodes, or “terms,” namely, “molecular function,” “cellular component,” and “biological process”; the majority of gene products in yeast are annotated to more specific (i.e., descendant) terms. We calculated the enrichment of these per-metabolite-class gene lists for all possible GO “biological process” terms using the hypergeometric distribution. Let 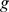 be the number of class-associated genes, 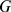 the number of genes in the genome, 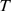 the number of genes in a GO term, and 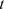 the number of class-associated genes that are also in the GO term. The p-value is then given by:
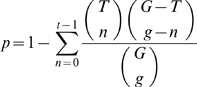
where 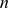 iterates from 0 to 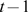. This equation therefore yields one minus the probability of observing 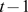 or fewer class-associated genes belonging to a given GO term, or equivalently, the probability of observing 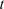 or greater class-associated genes belonging to that GO term.

These enrichment p-values were then Bonferroni corrected (i.e., 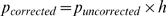, where 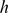 is the number of tests). Since only terms containing at least one of the significantly-correlated genes were tested for enrichment, the number of hypotheses tested 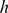 was 138 for the “TCA cycle” class, 611 for the “amino acids” class, 468 for the “glycolysis and pentose-phosphate pathway” class, and 620 for the “biosynthetic intermediates” class. All significant (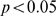) enrichments are given in Table 1.

### Gold Standard Construction

We assembled a “gold standard,” or a set of positive and negative examples of gene–metabolite interactions, from the Kyoto Encyclopedia of Genes and Genomes (KEGG) pathway database [33]. In order to find positive examples for the metabolite classes “amino acids” and “biosynthetic intermediates,” for each distinct pathway (e.g., “arginine and proline metabolism”) as defined by KEGG, the set of reactions in a pathway was collected and then matched to the enzymes that catalyzed these reactions. To generate gene–metabolite pairs, every measured metabolite that appeared in that pathway was then paired with this set of enzymes. For example, in the pathway “arginine and proline metabolism,” arginine, ornithine, and proline are all paired with all of the enzymes involved in the catabolism and biosynthesis of arginine and proline, including arginase (*CAR1*), ornithine-oxo-acid transaminase (*CAR2*), and proline oxidase (*PUT1*).

In the case of the “TCA cycle” and “glycolysis and pentose-phosphate pathway” metabolite classes, a similar procedure was used. However, compounds from both of these classes are used as carbon skeletons for a wide variety of metabolites. Therefore, to improve the specificity of these positive examples, positive examples for the “TCA cycle” class were drawn only from the list of reactions in the “TCA cycle” pathway, and positive examples for the “glycolysis and pentose-phosphate pathway” class were drawn only from “glycolysis and gluconeogenesis” and “pentose-phosphate pathway.” Additionally, to properly capture the structure of glycolysis and the pentose-phosphate pathway, each of these KEGG pathways was divided into two separate subpathways: these subpathways were upper and lower glycolysis (genes and metabolites upstream and downstream of fructose-1,6-bisphosphate, respectively, with FBP itself belonging to upper glycolysis), and the oxidative and non-oxidative branches of the pentose-phosphate pathway. Matching of metabolites within a pathway to reactions and to enzymes was performed in the same way as above (because of the structure of KEGG, this included certain enzymes outside the pathway that directly acted on one of the metabolites in these pathways, such as *ILV6*).

Certain “distributor” metabolites (2-oxoglutarate, acetyl-CoA, ADP, AMP, ATP, L-glutamate, L-aspartate, L-glutamine, NAD^+^, and NADP^+^) were excluded from the gold standard because they are common reactants or products in a very large number of reactions. For each metabolite class, 50 times as many random gene–metabolite pairs (drawn from outside the positive example set for all metabolite classes) were picked as negative examples, so that the final gold standard was 1.96% positives and 98.04% negatives (Dataset S2).

### Data Processing

In order to perform Bayesian integration, we first calculate the Pearson correlation of every metabolite and gene separately for each experimental perturbation. In order to ensure that these correlations are comparable between conditions, we enforce normality on the observed correlations 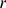 by applying a Fisher transform:
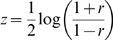
The resulting distribution 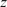 is then centered by the mean 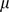 and divided by the standard deviation 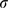 (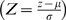). This process transforms the correlation distributions observed under nitrogen and under carbon starvation to be approximately equal to a normal distribution centered around zero, with a standard deviation of one. The Z-scores are then discretized into five bins; bin edges were 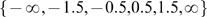, so that the “strong inverse” bin contained Z-scores more than 1.5 standard deviations below the mean, the “weak inverse” bin contained Z-scores from 0.5 to 1.5 standard deviations below the mean, the “no relationship” bin contained Z-scores 0.5 standard deviations above or below the mean, and so forth. These discretized data become the input for the Bayesian networks described below.

### Bayesian Network Training and Evaluation

The algorithm for finding gene–metabolite interactions is based on the Bayesian network shown in Figure 3. This network, whose structure is depicted in Figure 3B, relates the correlations observed between a gene and metabolite under each condition to (1) whether the gene and metabolite are related and (2) the class of the metabolite. More rigorously, this network specifies that, for a given gene 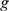 and metabolite 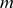, the discretized correlations observed under nitrogen starvation (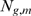) and under carbon starvation (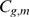) are dependent on the class (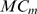) of the metabolite and whether or not the gene and metabolite are functionally related (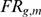). This network is therefore parametrized by the conditional probability distributions 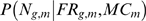 and 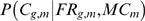, along with the prior probability of a gene–metabolite relationship 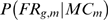, which simply reflects the proportion of positive and negative examples in our gold standard for each metabolite class (see above). The conditional probability distributions 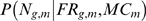 and 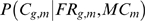 were calculated from the data using maximum likelihood [52]. In each of our examples, the value of every node is known, so this calculation reduced to counting the examples falling into each bin of correlation under nitrogen or carbon starvation for each possible value of 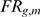 and 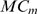. These counts were then divided by the total number of observations satisfying those values of 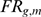 and 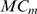 to yield probability distributions summing to one for 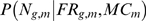 and 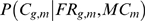.

After learning the parameters for this Bayesian network (shown in Figure 3C and 3D), we calculated the probability that a gene and metabolite were actually related given the observed correlations and the metabolite class, or 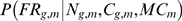. In our network, exact inference can be used to calculate 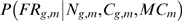:
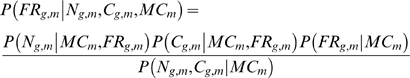
The numerator can be calculated directly from the learned parameters, and the denominator can be obtained by marginalization over 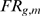.

We assessed this algorithm by generating a precision-recall curve, employing three-fold cross-validation to ensure unbiased evaluations. The gold standard was divided into random thirds; the network was then trained on two-thirds of the examples and evaluated on the remainder. This training was repeated three times, each time holding out a different third of the gold standard. Histograms of the confidence scores received by the positive and negative examples in the Bayesian integration process reveal that the positive examples from our gold standard indeed have significantly higher scores (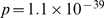 by Kolmogorov-Smirnov test), and can be found throughout the top predictions (Figure S1).

Our Bayesian network was trained and evaluated using the Bayes Net Toolbox for MATLAB [53].

### Literature-Based Evaluation of Top Predictions

The top 788 gene–metabolite pairs were predicted to be related by the Bayesian network with equal confidence. These top predictions were compiled and the pairs in the gold standard were removed; this yielded 764 predicted pairs. We then added 250 random gene–metabolite pairs, and analyzed the random and predicted sets together. This analysis was performed blind to whether pairs were predicted by the algorithm or randomly selected. The predictions were evaluated based on four categories:

1. *Specific GO function*. A pair received a point if a GO term to which the gene was annotated contained the name of the metabolite or metabolite class, and this GO term was more specific than a term in the GO Functional Slim [54] list.
2. *Specific TF target*. If the gene in question had an upstream binding site (according to Harbison et al. [55] or Tachibana et al. [56]) for a transcription factor known to regulate a specific branch of metabolism (for example, methionine and a gene with a *MET4* site, or a sulfur-containing amino acid and a gene with a *CBF3* site), then the gene–metabolite pair received a point for each binding site.
3. *Specific documented interaction*. A pair received a point if a Pubmed search with each member of the pair as a search term was able to reveal a confirmed interaction between the two, as in FBP and *VID24*.
4. *Relevant knockout phenotype*. A pair received a point if there was a documented knockout (KO) phenotype for the gene in question listed on the Saccharomyces Genome Database (SGD) [57] that related to the metabolite in question, such as failure of the knockout to grow in media not supplemented with that metabolite.

Since relatively few genes and metabolites have been studied for interactions, we expect that the gene–metabolite pairs scored according to this evaluation will contain many false negatives, or gene–metabolite pairs for which there is no evidence simply because the relationship between those particular genes and metabolites have not yet been studied, despite the presence of a functional interaction.

### Media Composition

“YNB” minimal media consisted of 6.7 g yeast nitrogen base without amino acids and 20 g D-glucose per 1 L. “YNB-C” carbon starvation media consisted of 6.7 g yeast nitrogen base without amino acids per 1 L, with no glucose. “YNB-N” minimal media consisted of 6.7 g yeast nitrogen base without amino acids and without ammonium sulfate and 20 g D-glucose per 1 L. 30 g of three-times-washed ultrapure agarose was added per 1 L to make agarose plates.

## Supporting Information

Dataset S1 Transcript data. Transcriptional data, expressed as log2 ratios to time zero, for 10, 30, 60, 120, 240, and 480 minutes post-induction of nitrogen starvation (removal of ammonium) or carbon starvation (removal of glucose).
(2.20 MB XLS)

Dataset S2 Gold standard. Set of positive and negative examples of gene-metabolite interactions used to train the Bayesian network, assembled from the KEGG (Kyoto Encyclopedia of Genes and Genomes) Pathway database.
(0.21 MB XLS)

Dataset S3 Literature study results. This table is a representation of the described blind literature study, in which the 764 top predictions (that were not in the gold standard) were scored together with 250 random gene-metabolite pairs.
(0.25 MB XLS)

Figure S1 Distribution of prediction scores. This figure shows histograms of the confidence scores (x-axis) from the Bayesian integration procedure for negative (dashed light gray) and positive (solid dark gray) examples in the gold standard. The plot reveals that the distribution of positive pairs shows a propensity for higher scores (p = 1.1×10^−39^, by Kolmogorov-Smirnov test) and that the distribution of positive pairs is smooth.
(0.02 MB PDF)

Figure S2 Enlarged plots of selected metabolite versus gene concentrations under nitrogen starvation. Because concentrations of the glycolytic metabolites hexose-phosphate and phosphoenolpyruvate had a smaller dynamic range under nitrogen starvation than under carbon starvation, the first five examples of metabolite vs. transcript concentration plots in the nitrogen starvation condition from Figure 2 have been plotted with an expanded x-axis.
(0.01 MB PDF)

Figure S3 Comparison of zero timepoints from metabolomic data shows robustness to biological and technical variation. Since we have two independent measurements of metabolite counts in unperturbed cells (the zero timepoints in the carbon starvation and in the nitrogen starvation experiments), these measurements can be compared to assess the technical and biological reproducibility. The agreement between the time points is very high (y = 1.03×, R^2^ = 0.998). We also calculated Lin's concordance coefficient, which is a normalized measure of the distance from the 45° line through the origin y = x, where a score of 0 would be totally non-reproducible and a score of 1 would be identical; this value was calculated to be 0.98, indicating very high reproducibility.
(0.02 MB PDF)
