# Supplementary figures and images for: Coordinated Concentration Changes of Transcripts and Metabolites in Saccharomyces cerevisiae
Source: PLoS Comput Biol. 2009 Jan 30;5(1):e1000270. doi: 10.1371/journal.pcbi.1000270 (PMC2614473; doi:10.1371/journal.pcbi.1000270)

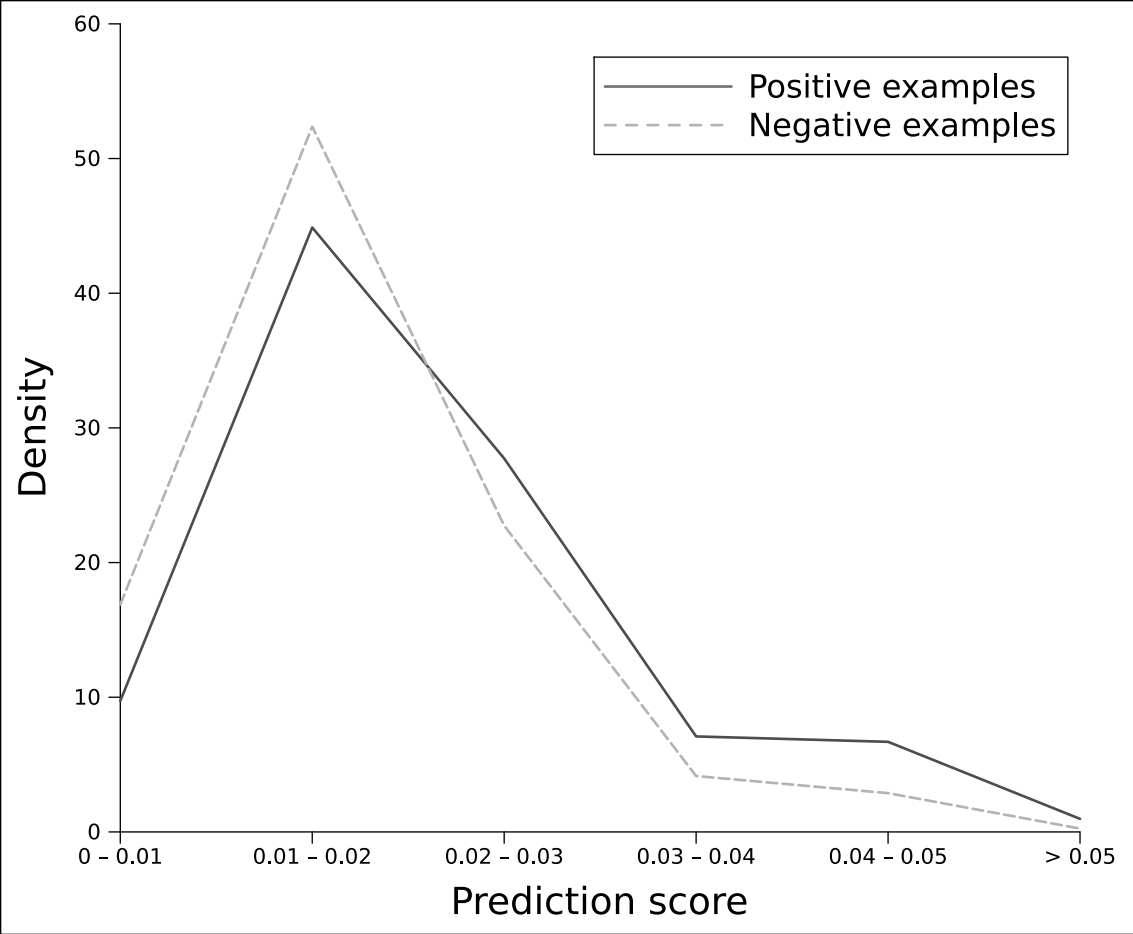

Supplement: Figure S1 — Distribution of prediction scores. This figure shows histograms of the confidence scores (x-axis) from the Bayesian integration procedure for negative (dashed light gray) and positive (solid dark gray) examples in the gold standard. The plot reveals that the distribution of positive pairs shows a propensity for higher scores (p = 1.1×10^−39^, by Kolmogorov-Smirnov test) and that the distribution of positive pairs is smooth. (0.02 MB PDF) [file pcbi.1000270.s004.pdf]

# Reproducibility of metabolite measurements

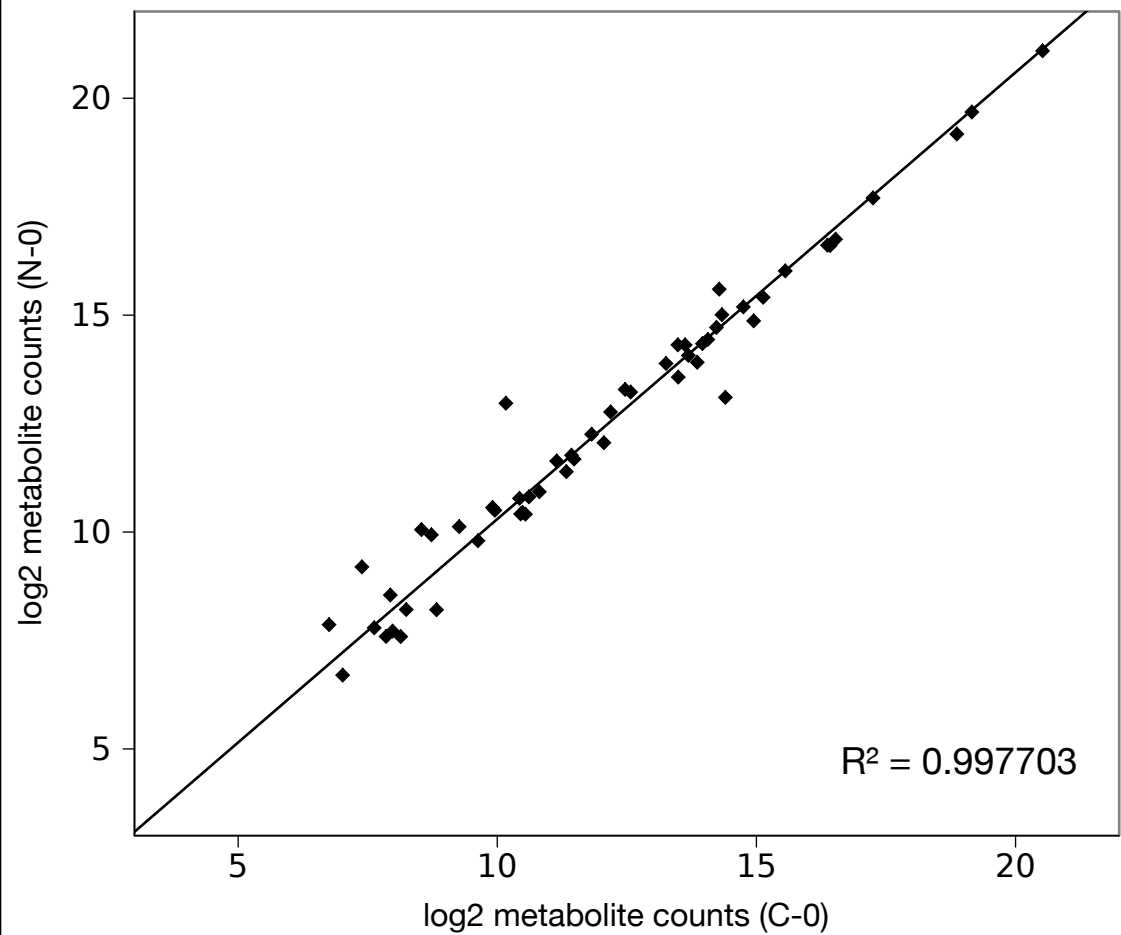

Supplement: Figure S3 — Comparison of zero timepoints from metabolomic data shows robustness to biological and technical variation. Since we have two independent measurements of metabolite counts in unperturbed cells (the zero timepoints in the carbon starvation and in the nitrogen starvation experiments), these measurements can be compared to assess the technical and biological reproducibility. The agreement between the time points is very high (y = 1.03×, R2 = 0.998). We also calculated Lin's concordance coefficient, which is a normalized measure of the distance from the 45° line through the origin y = x, where a score of 0 would be totally non-reproducible and a score of 1 would be identical; this value was calculated to be 0.98, indicating very high reproducibility. (0.02 MB PDF) [file pcbi.1000270.s006.pdf]
