# Supplementary material for: Coordinated Concentration Changes of Transcripts and Metabolites in Saccharomyces cerevisiae
Source: PLoS Comput Biol. 2009 Jan 30;5(1):e1000270. doi: 10.1371/journal.pcbi.1000270 (PMC2614473; doi:10.1371/journal.pcbi.1000270)
Supplement: Figure S2 — Enlarged plots of selected metabolite versus gene concentrations under nitrogen starvation. Because concentrations of the glycolytic metabolites hexose-phosphate and phosphoenolpyruvate had a smaller dynamic range under nitrogen starvation than under carbon starvation, the first five examples of metabolite vs. transcript concentration plots in the nitrogen starvation condition from Figure 2 have been plotted with an expanded x-axis. (0.01 MB PDF) [file pcbi.1000270.s005.pdf]

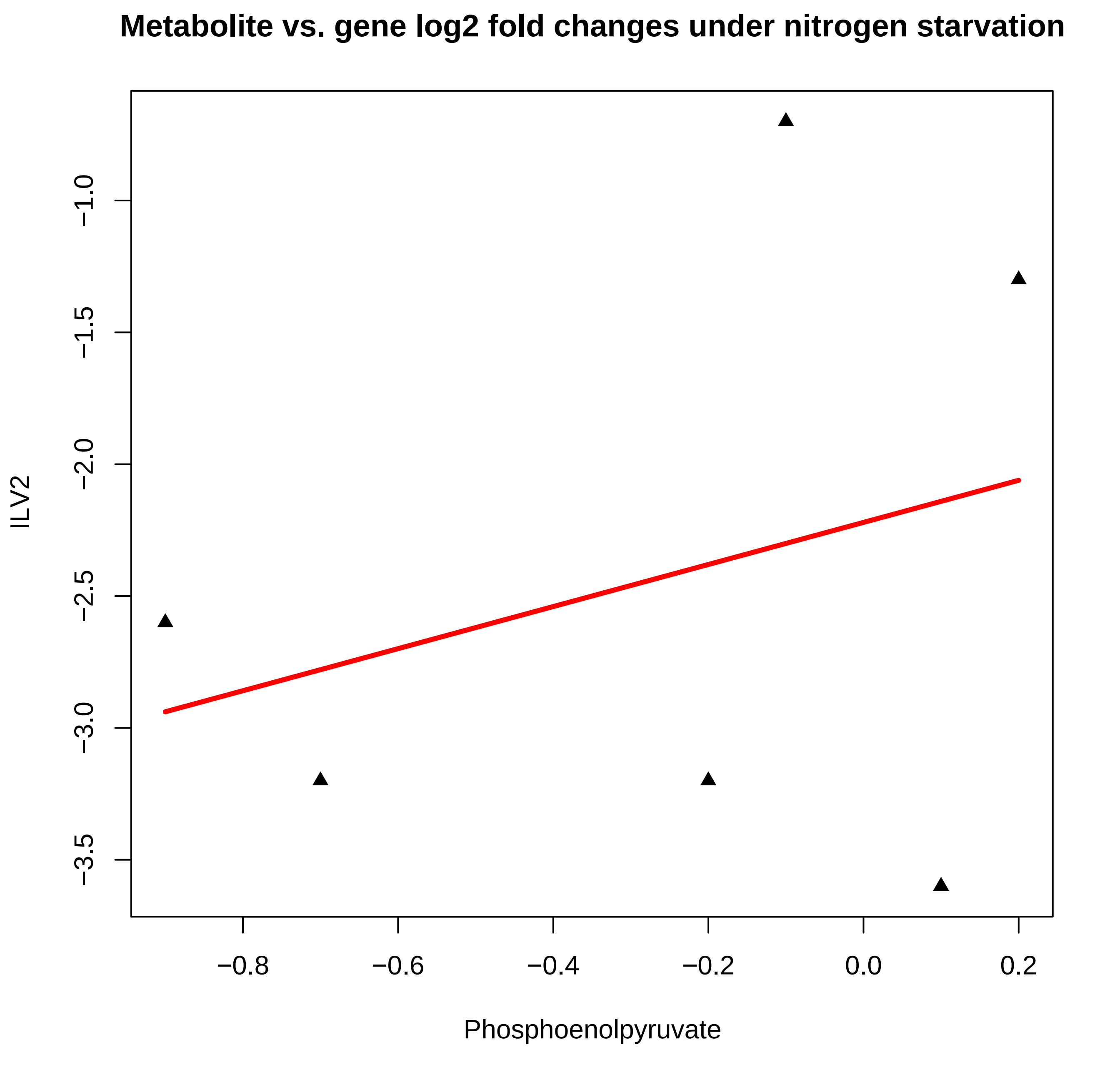

Metabolite vs. gene log2 fold changes under nitrogen starvation
−1.0
−1.5
−2.0
−2.5
−3.0
−3.5
ILV2
−0.8
−0.6
−0.4
−0.2
0.0
0.2
Phosphoenolpyruvate

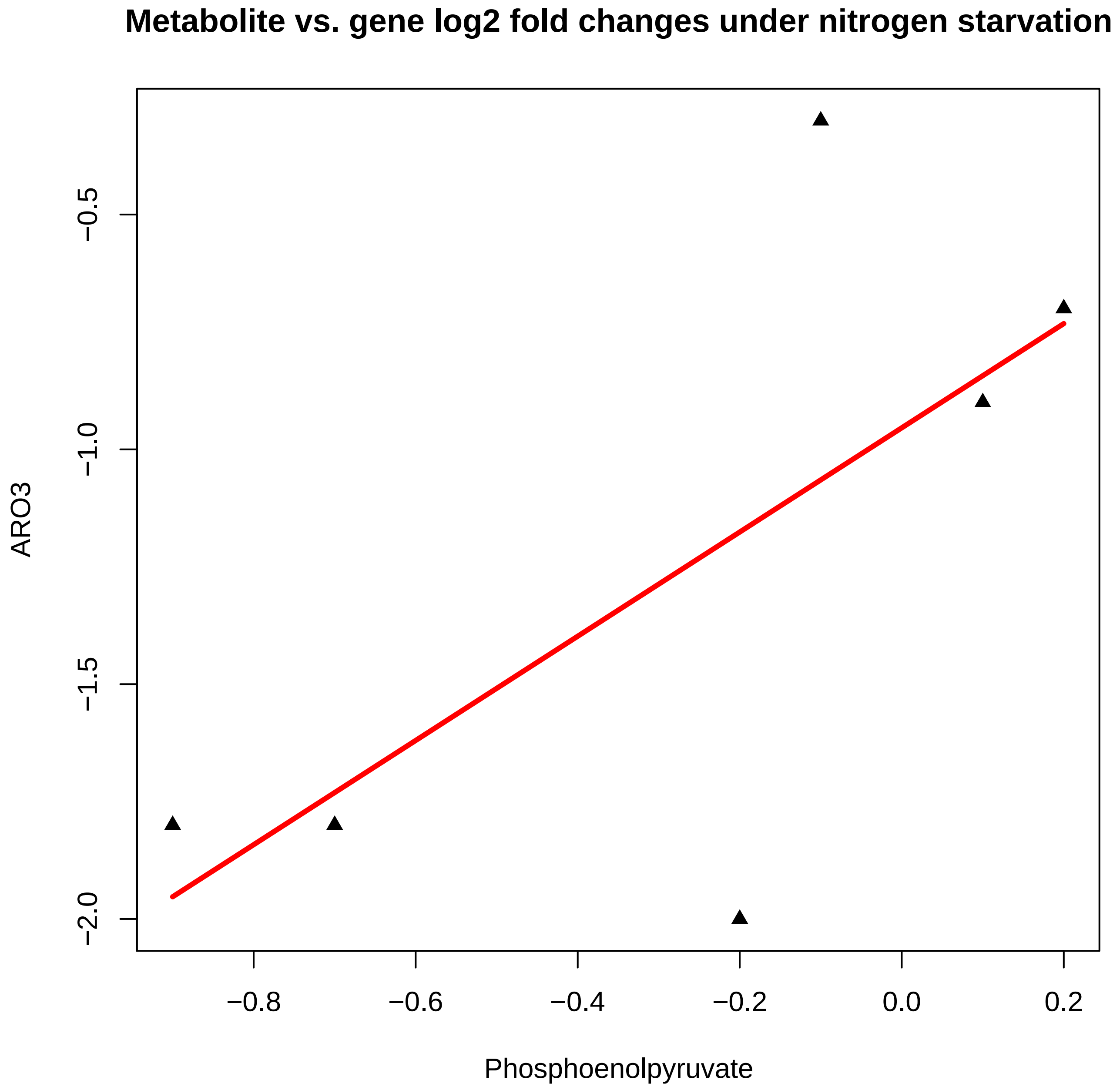

Metabolite vs. gene log2 fold changes under nitrogen starvation
ARO3
−0.5
−1.0
−1.5
−2.0
−0.8
−0.6
−0.4
−0.2
0.0
0.2
Phosphoenolpyruvate

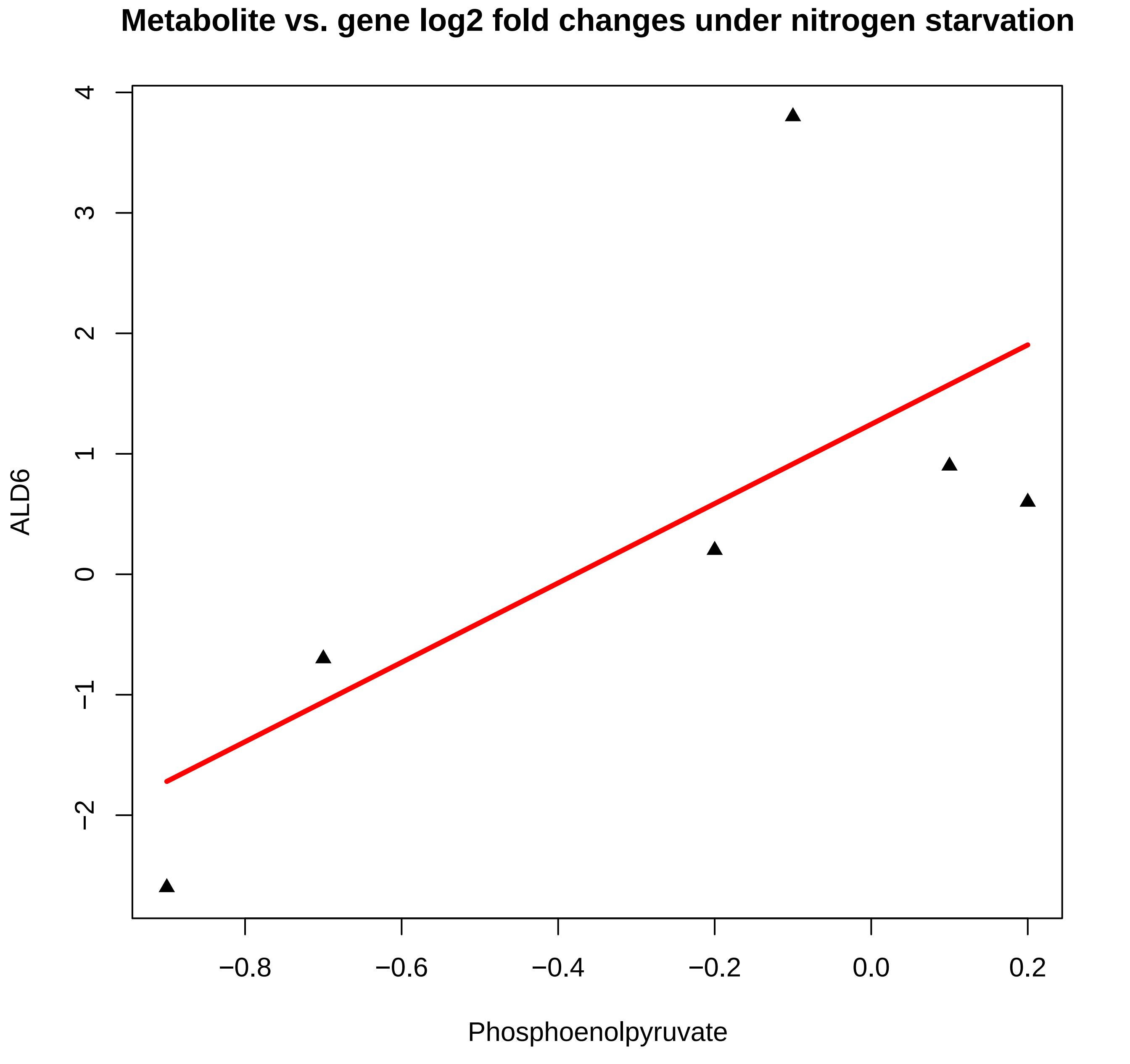

Metabolite vs. gene log2 fold changes under nitrogen starvation
ALD6
Phosphoenolpyruvate
−0.8
−0.6
−0.4
−0.2
0.0
0.2
−2
−1
0
1
2
3
4

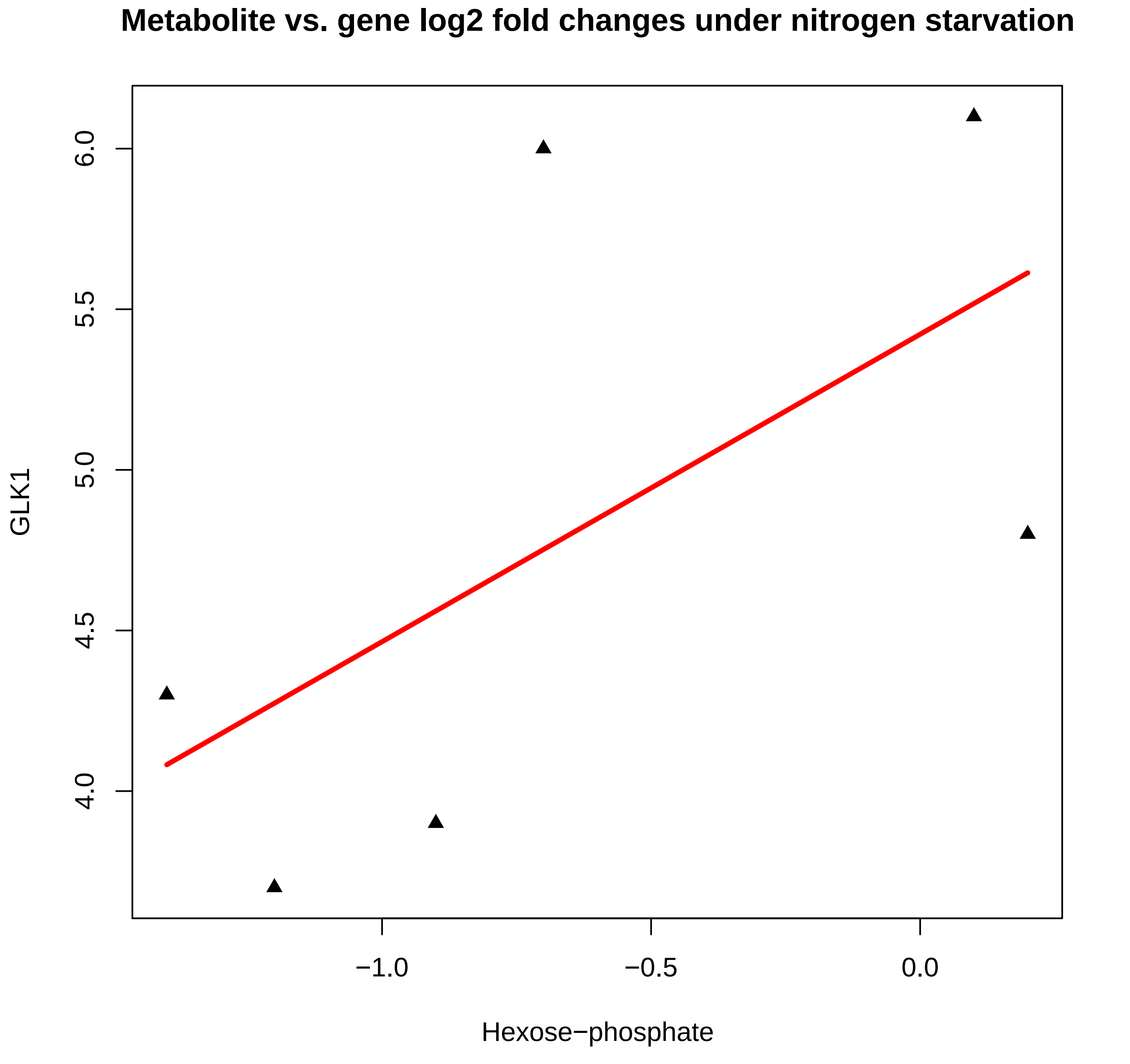

Metabolite vs. gene log2 fold changes under nitrogen starvation
GLK1
6.0
5.5
5.0
4.5
4.0
−1.0
−0.5
0.0
Hexose−phosphate

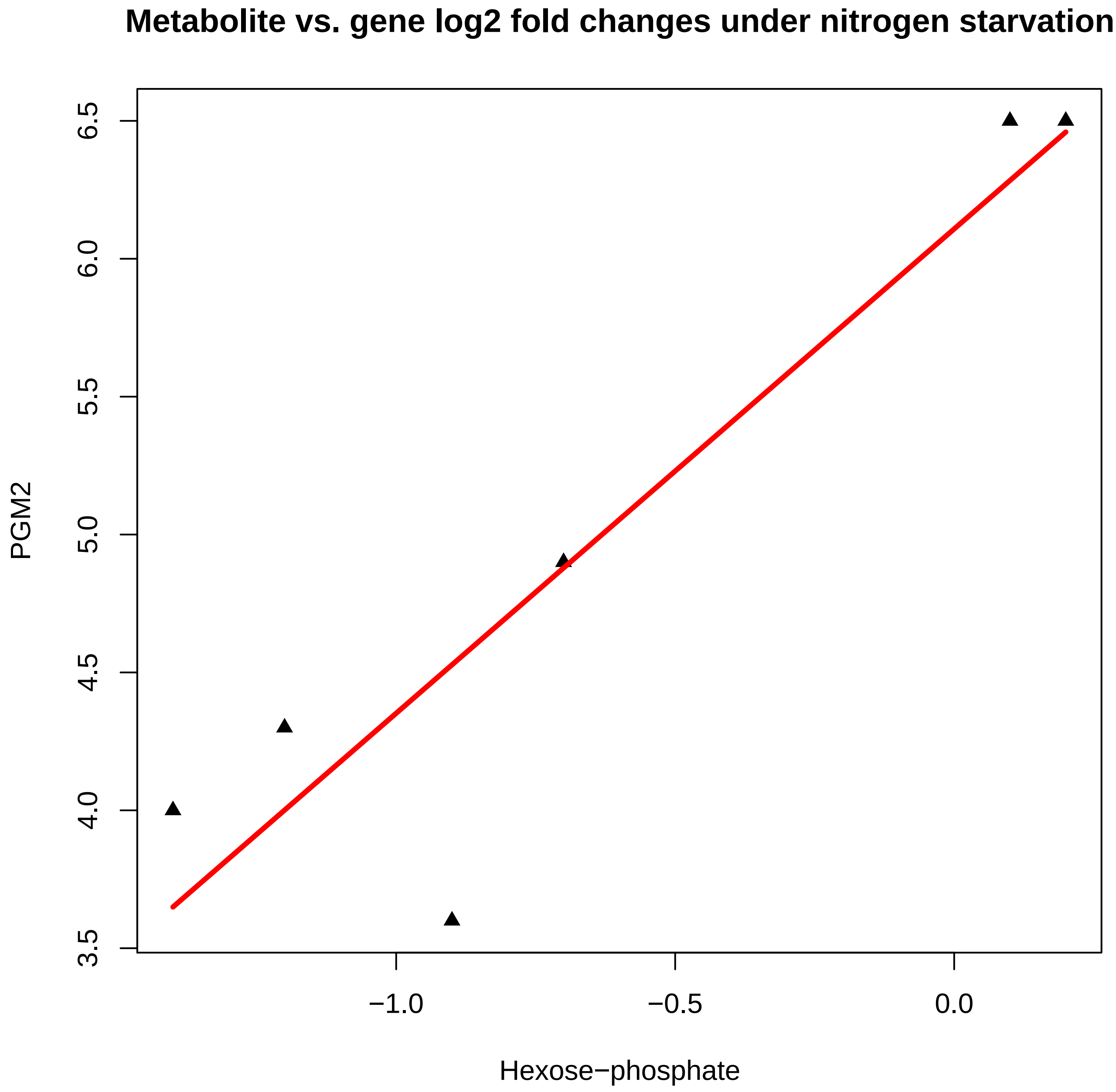

Metabolite vs. gene log2 fold changes under nitrogen starvation
PGM2
6.5
6.0
5.5
5.0
4.5
4.0
3.5
−1.0
−0.5
0.0
Hexose−phosphate
